# Active digital spoof plasmonics

**DOI:** 10.1093/nsr/nwz148

**Published:** 2019-10-04

**Authors:** Hao Chi Zhang, Tie Jun Cui, Yu Luo, Jingjing Zhang, Jie Xu, Pei Hang He, Le Peng Zhang

**Affiliations:** 1 State Key Laboratory of Millimeter Waves, Southeast University, Nanjing 210096, China; 2 The Photonics Institute and Centre for Optoelectronics and Biophotonics, School of Electrical and Electronic Engineering, Nanyang Technological University, Singapore 639798, Singapore

**Keywords:** digital coding, digital modulation, plasmonics, digital metamaterials

## Abstract

Digital coding and digital modulation are the foundation of modern information science. The combination of digital technology with metamaterials provides a powerful scheme for spatial and temporal controls of electromagnetic waves. Such a technique, however, has thus far been limited to the control of free-space light. Its application to plasmonics to shape subwavelength fields still remains elusive. Here, we report the design and experimental realization of a tunable conformal plasmonic metasurface, which is capable of digitally coding and modulating designer surface plasmons at the deep-subwavelength scale. Based on dynamical switching between two discrete dispersion states in a controlled manner, we achieve digital modulations of both amplitude and phase of surface waves with nearly 100% modulation depth on a single device. Our study not only introduces a new approach for active dispersion engineering, but also constitutes an important step towards the realization of subwavelength integrated plasmonic circuits.

## INTRODUCTION

Digital/information science focuses on the effective collection, storage, retrieval, and use of information. In general, the cornerstones of the digital science are binary coding (designing two distinct physical states with significantly different system responses, denoted as ‘0’ and ‘1’) and modulation (dynamically manipulating the ‘0’ and ‘1’ states in the time domain to transfer information). Based on these technologies, the signals (carried by the electric currents or electromagnetic waves) can be extracted, transmitted and processed in a controlled manner.

On the other hand, metamaterials, subwavelength artificial structures, have been widely applied to the control of electromagnetic (EM) fields or light waves [1–6], through their access to various unusual material parameters, such as negative [7] and near-zero [8] refractive indices and extremely strong chirality [9]. Such a concept has also been applied to plasmonics [10–12] to manipulate the dispersion state of surface waves at low frequencies, so as to spoof natural surface plasmons (SPs) while suppressing the metallic loss [13]. Due to their distinctive properties of strong field confinement and enhancement, spoof plasmonic metamaterials promise useful applications in low-cross-talk waveguides [14], miniaturized sensors [15], and so on [16–18]. However, because of their analog nature and immutable functionality, spoof plasmonic metamaterials are not compatible with traditional digital technology, making digital control of surface waves still difficult even at low frequencies.

Recently, digital technology was introduced to metamaterials [19]. Unlike electronic circuits, which use two-bit voltage to code information, digital metamaterials use two-bit structures (i.e. two different coding unit cells with opposite reflection phases) to control the reflection, transmission, and deflection of light [20–24]. Such a technique can be applied to both the space [25] and time domains [26] to engineer the wavefront of light at not only the fundamental but also the harmonic frequencies. However, the digital metamaterial concept has thus far been limited to the control of spatial light because the anti-permittivity or anti-phase techniques cannot be directly extended to the subwavelength scale to control surface plasmons.

**Figure 1.**
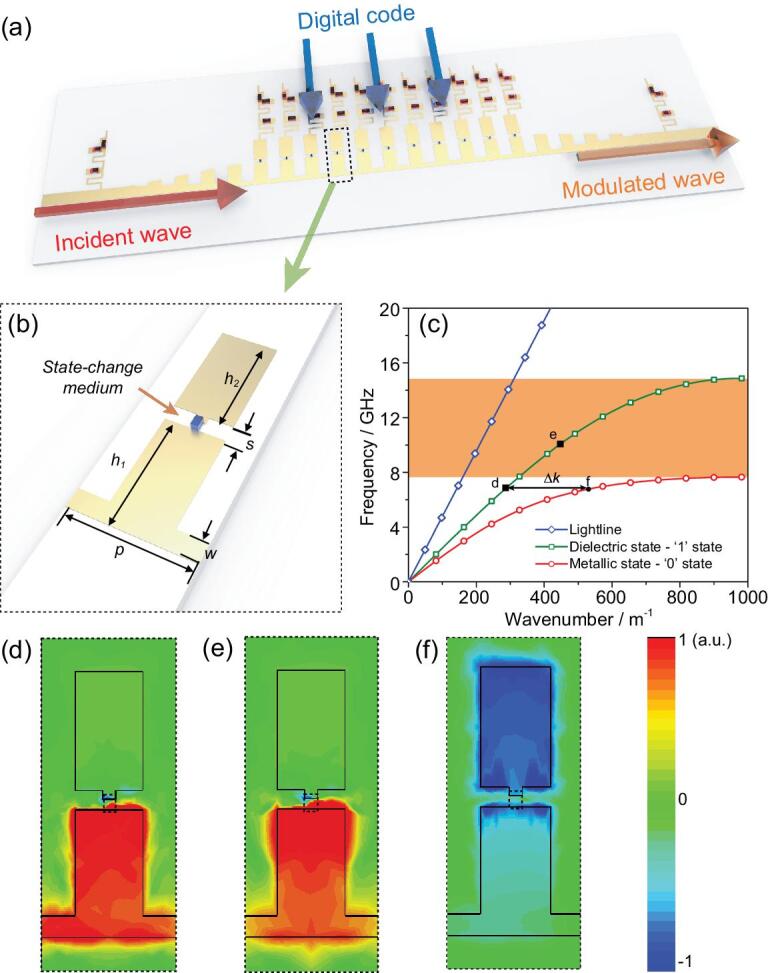
A schematic diagram, dispersion properties and eigenmode field distributions of the digital SPs. (a) A schematic diagram of the prototype of the digital SP waveguide, which is supported by the direct interaction between the incident wave and digital code. (b) A schematic diagram of a single digital SP unit, which is described by the period *p* = 3.2 mm, strip width *w* = 0.5 mm, slit width *s* = 0.45 mm, height of the lower metallic bar *h*_1_ = 3 mm, and height of the upper metallic bar *h*_2_ = 2.8 mm. (c) Simulated dispersion curves of the digital SP unit with different states. (d–f) Eigenmode field distributions of the digital SP units with ‘1’ state (d, e) and ‘0’ state (f) at 7.2 GHz (d, f) and 10 GHz (e).

Here, we propose a new approach based on dynamic dispersion engineering to realize digital spoof surface plasmons. The digital spoof plasmonic metamaterials are composed of judiciously designed surface metallic patterns and state-change medium (e.g. phase change materials or PIN diode), as shown in Fig. 1a and b, respectively. The dynamic engineering of dispersion states is demonstrated analytically and observed experimentally, based on the transmission spectrum and near-field distributions. We first use the transmission spectrum and near-field measurement to demonstrate the feasibility of the dynamic surface plasmon polariton (SPP) dispersion engineering with designed structure and state-change medium, and then realize a prototype structure to implement both amplitude and phase modulations (i.e. amplitude shift keying (ASK) and phase shift keying (PSK)) on a single sample. Compared to traditional analog devices such as phase shifters, the proposed designer SP waveguide has two major advantages: 1. it supports subwavelength hybrid plasmon modes, and therefore presents lower cross-talk and can more easily couple to free-space radiation; 2. it is a multifunctional device that can work as not only a phase shifter but also a tunable filter or switch, and therefore provides greater freedom for practical applications, such as anti-interference. Hence, the proposed digital designer SPs enable signal processing based on a single medium, a task that is extremely difficult in traditional information technology and that has not been discussed in previous research on digital metamaterials [27,28].

## RESULTS

### Digital designer SPs and realization

Manipulation of dispersion, the macroscopic property of the interaction between metamaterial structures and incident EM waves, provides a direct means to engineer the transmission behavior of EM waves. Inspired by this idea, the dispersion states of designer SPs are chosen to realize the metallic (‘0’ state) and dielectric (‘1’ state) states using state-change medium made of semiconductor [29,30], phase-change material [31,32] or microfluidics [33]. A structure supporting digital designer SPs, as sketched in Fig. 1b, is constructed by a deep-subwavelength-scale state-change medium (here, without loss of generality, it is selected as the PIN diode and its volume is smaller than 5 × 10^−7^λ^3^) and surrounding planar designer SP structures. The highly confined nature of the surface modes makes the whole device extremely sensitive to tiny changes in the background. This property greatly enhances the modulation efficiency of the designer SP devices. Through finite-element analysis of the eigenmode (detailed information can be found in the supplementary information (SI) online) [23], the dispersion curves of digital designer SPs with different states are displayed in Fig. 1c, where the metal and dielectric substrate are selected as copper and Rogers RT 5880, respectively, and detailed geometrical parameters of the designer SPP structure are included in the SI. To accelerate the convergence in the finite-element simulation, the ‘0’ and ‘1’ states of the state-change medium are considered as the perfect electrical conductor (PEC) and lossless dielectric with the relative permittivity *ε_r_* = 18.5 (i.e. the permittivity of the actual packaging material of the PIN diode), respectively. When the loss is small, this approximation only leads to small deviations to dispersion curves, as verified by previous works [14,17,24,34–36]. Moreover, we would like to highlight that the controlling voltage will not significantly modify the dispersion curves of the digital SPs as long as the state of the state-change medium remains unchanged. It is also worth mentioning that the effect of the bias voltage port can be minimized by introducing ambient circuits (see the SI). Hence, the digital coded voltage port can be ignored in the eigenmode analysis.

The dispersion diagram demonstrates that the dispersion curves of both ‘0’ and ‘1’ states deviate gradually from the light line in vacuum, and then asymptotically approach two different cut-off frequencies, behaving like natural SP modes at optical frequencies. From the physical view, the subwavelength-scale state-change medium controls the gorge between the upper and lower metallic slices to maximize the modulation depth, which is defined as the ratio between the difference and sum of transmission ratio of the ‘1’ and ‘0’ states. When the state-change medium behaves as a metallic state (‘0’ state), the gorge allows the high-frequency current through. Hence, the surface plasmonic resonance occurs around the surface of the whole structure, leading to a cut-off frequency of about c/4(*h_1_* + *h_2_*), as shown in Fig. 1f. It is worth noting that the field in this figure is distributed in both of the upper and lower metallic slices. In contrast, when the state-change medium behaves as a dielectric state (‘1’ state), the high-frequency current is interdicted in the gorge, resulting in the partial resonance only in lower metallic slices, whose cut-off frequency is approximated as c/4*h_1_*, as shown in Fig. 1d and e. Figure 1d and e are both with state ‘1’, where the corresponding resonances are localized in the lower metallic slices with difference phases. On the other hand, Fig. 1f is state ‘0’ and possesses different resonance condition and mode profile. Hence, the digital designer SPs allow us to customize dispersion curves of both ‘0’ and ‘1’ states, independently, through changing the geometrical parameters *h_1_* and *h_2_*.

### Dynamic dispersion engineering

**Figure 2.**
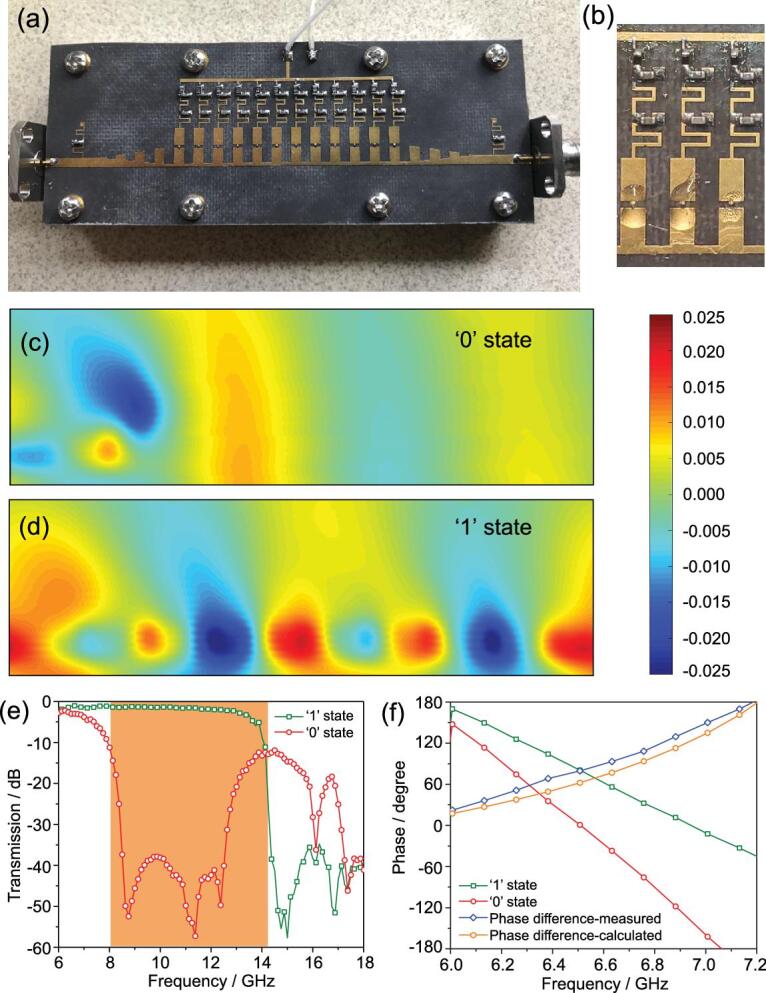
Photographs of the fabricated prototype and measured results of the digital designer SPs. (a) A macroscopic view of the fabricated prototype. (b) An enlarged view of the fabricated prototype. (c, d) The measured near-field distributions of the digital SP waveguide with ‘0’ (c) and ‘1’ (d) states at 9 GHz. (e, f) The measured transmission and phase spectra of the digital SP waveguide with ‘0’ (e) and ‘1’ (f) states.

The digital SPs provide a logical approach to engineer the dispersion state through switching the ‘0’ and ‘1’ states of the state-change material. To realize this idea, we construct and fabricate a digital SP waveguide by arranging the SP units with period *p* along the *x* axis on the top surface of a dielectric substrate made by Rogers RT 5880 (see the section entitled ‘Method’) and metallic ground on the bottom surface of the substrate as shown in Fig. 2a and b, where PIN diodes, MA4AGBL912, and the ambient circuits act as the state-change medium controlled by digital coded voltage and the chokes stop the high-frequency energy leaking to the bias port and metallic ground (see Fig. S2b in SI), respectively. Moreover, two momentum compensations are especially designed to reduce the mismatch between the transverse electromagnetic (TEM) or quasi-TEM mode and the SP modes using a planar gradient index structure (see Fig. S2c in SI) [35–38]. To verify the performance of the digital SP waveguide, the transmission spectra and near-field distributions are measured by a vector network analyzer (VNA, Agilent N5230C) and near-field scanning mapper (see the section entitled ‘Methods’), respectively, as shown in Fig. 2c–f. We observe that the EM wave was truncated by the first digital SP unit behaving as a ‘0’ state, and cannot propagate through the digital SP array, as displayed in Fig. 2c. The truncated behavior is also quantitatively verified in Fig. 2e, which implies that only less than 1‰ of EM energy can go through the digital SP waveguide from 8.4 to 12.6 GHz. In contrast, the incident EM wave can propagate along the digital SP waveguide behaving as a ‘1’ state without reflection, which is also demonstrated by the spectrum measurement result in Fig. 2e. More than 80% of the EM energy can be received by the output port, and the transmittance of the signal can be improved by using low-loss dielectric materials and state-change media [39]. Note that the truncating frequencies of these two states are identical to the dispersion analysis in Fig. 1c.

Hence, the dispersion control based on the digital designer SPs provides an approach to a significant transmission ratio between opening and closure states. In other words, the digital designer SPs allow us to manipulate two distinct propagation states based on dispersion engineering. If the frequency of the incident wave is lower than the cut-off frequency of the ‘0’ state (i.e. 8 GHz in our design), the amplitude states of the ‘0’ and ‘1’ states cannot be engineered by changing the digital states because both ‘0’ and ‘1’ states behave as the transmission state, as shown in Fig. 1c. Nevertheless, in this frequency band, the inherent wavenumber difference between the ‘0’ and ‘1’ states can be accumulated along the propagation direction, leading to a phase difference between the ‘0’ and ‘1’ states, as shown in Fig. 2f. Note that this digital designer SP waveguide can provide a 180-degree phase difference at 7.2 GHz, which can be engineered by changing the number of SPP units in the propagation direction and can match the calculated result of dispersion curves well, indicating that the output EM signals are opposite to each other. Hence, the phase shift level of the digital designer SPs can also be manipulated by converting the digital states of the digital designer SPs owing to the ability of dispersion engineering and natural properties of SPs.

### Direct ASK and PSK modulations

Similar to the coding process in digital science, the dynamic dispersion-engineering ability of the digital designer SPs has been demonstrated to change the physical (amplitude and phase) states of the transmitted EM waves. Furthermore, the time-varying digital states of digital designer SPs are taken into consideration to demonstrate interaction between the time-variant digital signals and high-frequency EM waves, equivalent to the digital modulation (i.e. ASK and PSK) process in digital science.

**Figure 3.**
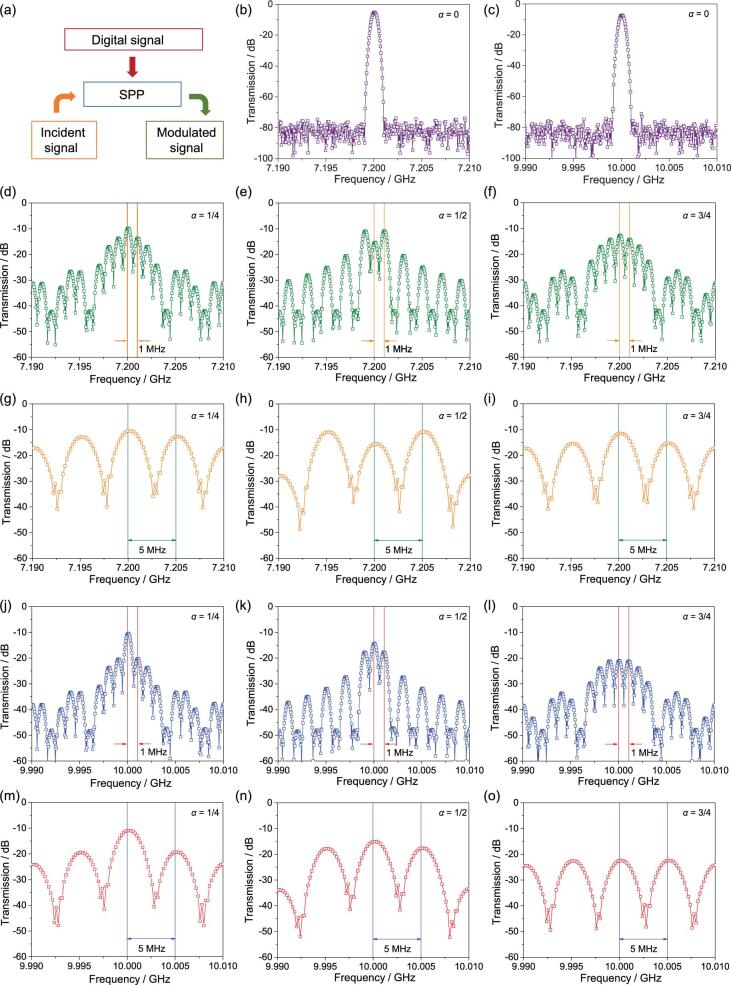
The measured non-linear spectra of transmitted signals of the digital designer SP waveguide with different incident signals, different digital signals, and different duty factors. (a) A schematic diagram of the direct digital modulation using the digital designer SPs. (b, c) The incident signals without modulations at 7.2 GHz (b) and 10 GHz (c). (d–f) The incident signals modulated by 1 MHz square wave signal at 7.2 GHz with 1/4 (d), 1/2 (e), and 3/4 (f) duty factors. (g–i) The incident signals modulated by 5 MHz square wave signal at 7.2 GHz with 1/4 (g), 1/2 (h), and 3/4 (i) duty factors. (j–l) The incident signals modulated by 1 MHz square wave signal at 10 GHz with 1/4 (j), 1/2 (k), and 3/4 (l) duty factors. (m–o) The incident signals modulated by 5 MHz square wave signal at 10 GHz with 1/4 (m), 1/2 (n), and 3/4 (o) duty factors.

Considering visualization, the time-variant digital signals are square wave signals with *f_m_* (it selected as the 1 or 5 MHz in experiment) and are input to each digital designer SP unit simultaneously to control the ‘0’ and ‘1’ states, as shown in Fig. 3a. To verify the digital modulation of the digital designer SPs, non-linear spectra with different cases are obtained using the non-linear spectrum measurement system, as sketched as in Fig. 3b–o. In these figures, firstly, the spectrum without digital modulation is displayed in Fig. 3b and c, which demonstrates that the output spectrum is also a pure spectrum without harmonics. Next, we introduce the digital coding stream into the digital designer SPs to control the dispersion state in real time and achieve the digital modulation of designer SPs, which can directly respond by the frequency spectrum of the *f_0_* + *nf_m_* (*f_0_* is the frequency of the incident wave, and *n* is the integer number; a detailed analysis can be found in the section entitled ‘Methods’). Considering the fact that the corresponding functions for different states depend on the incident frequency, it is necessary to analyze the modulation behavior of the digital designer SPs under different incident frequencies.

Based on the dispersion analysis, there is an inherent phase difference between the corresponding functions for the ‘0’ and ‘1’ states at lower frequencies (i.e. lower than the cut-off frequency of the ‘0’ state), as sketched in Fig. 2f. In particular, for the incident wave of 7.2 GHz, the inherent phase is exactly equal to 180 degrees, implying that the corresponding function for the ‘0’ and ‘1’ states can be selected as *β*exp[*i*(2π*f*_inc_*t* + *φ_0_*)] and exp[*i*(2π*f*_inc_*t* + *φ_0_* + π)] (*φ_0_* and *β* are the initial phase and inherent amplitude ratio of the ‘0’ state, respectively). Hence, the modulated result is identical to the PSK method, and the spectral energies of the modulated harmonics are discrete at *f*_inc_ + *k*/*T* and symmetric to the incident wave frequency and can be controlled by the duty ratio *α*, which can change the spectral width of the modulated signal, as shown in Fig. 3d–i. Meanwhile, for our case, the amplitude ratio *β* is about 0.8. Hence, the duty factor has only a little influence on the total spectral energy.

On the other hand, for the incident wave whose frequency is higher than the cut-off frequency of the ‘0’ state, the corresponding functions for the ‘0’ and ‘1’ states are 0 and exp[*i*(2π*f*_inc_*t* + *φ_0_*)] (*φ_0_* is the initial phase), respectively. Hence, this case is identical to the modulated result for the traditional ASK method. Observably, the spectral coefficients of the modulated transmitted signals, which can be controlled by the duty ratio *α*, are discrete at *f*_inc_ + *k*/*T* and symmetric to the incident wave frequency, as shown in Fig. 3j–o. However, unlike PSK modulation, the duty factors have a direct effect on the total spectral energy of ASK because transmission is almost blocked completely at the ‘0’ state. Hence, they play a more significant role (the transmission) in ASK than PSK modulation.

**Figure 4.**
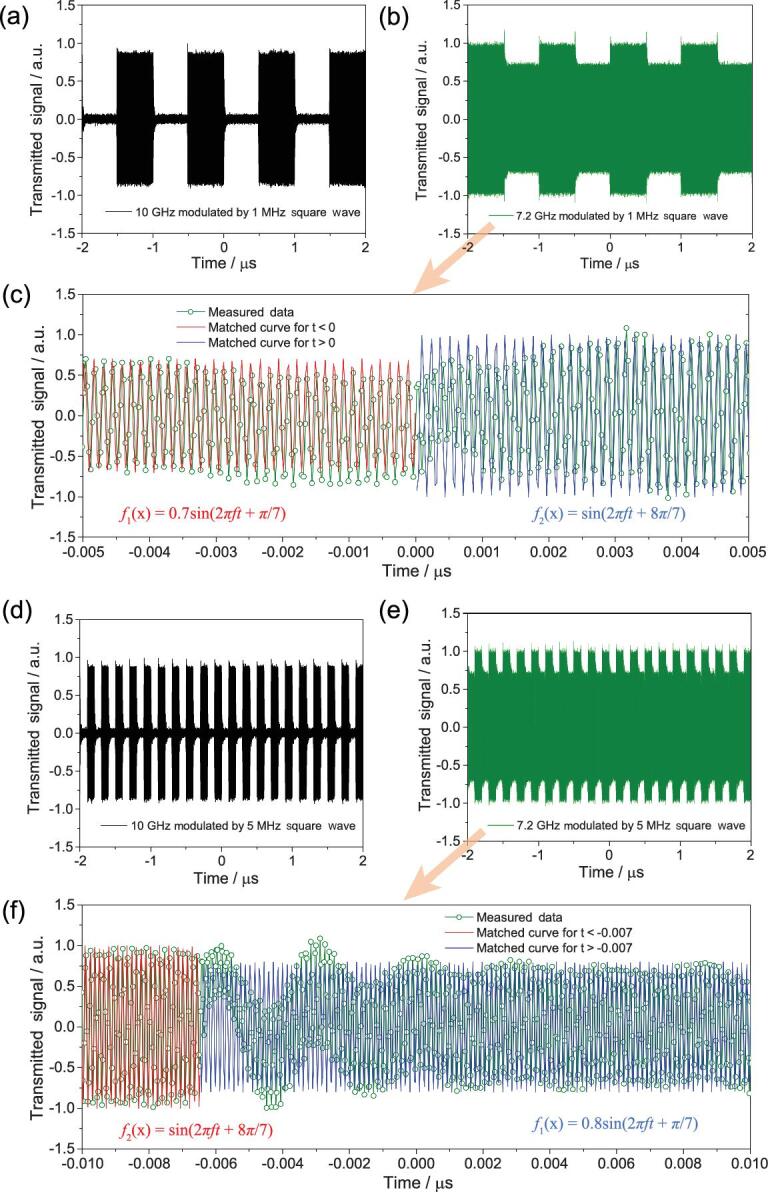
The measured time-domain spectra of the transmitted signal of the digital designer SP waveguide with different incident signals and different digital signals. (a, b) The incident signals at 10 GHz (a) and 7.2 GHz (b) modulated by 1 MHz square wave signal. (c) An enlarged view of Fig. 4b around 0 s. (d, e) The incident signals at 10 GHz (d) and 7.2 GHz (e) modulated by 5 MHz square wave signal. (f) An enlarged view of Fig. 4e around 0 s. The duty factors of square wave signal are 1/2 in this figure.

To verify the time-domain modulation ability of the digital designer SPs more intuitively, the time-domain spectroscopies of the digital designer SPs with PSK and ASK modulations are also shown in Fig. 4. In the ASK case, we can observe that the transmitted waveforms are deeply modulated by the 1 MHz or 5 MHz square wave, as shown in Fig. 4a and d, which displays a signification transmission ratio between the ‘0’ and ‘1’ states in the time domain as analyzed above. In the PSK case, the transmission ratios are much smaller than those of ASK, as shown in Fig. 4b and e, satisfying the precondition for the traditional PSK modulation. Meanwhile, Fig. 4c and f displays the enlarged view of the PSK time-domain spectra in a smaller time window (a detailed zoomed-in view can be seen in Fig. S3 in SI). It is noteworthy that the phases of the time-domain signals are reversed when switched between the ‘0’ and ‘1’ states for most parts of the time. Alternatively, a fluctuation in the amplitude and phase, resulting from charge and discharge of the electrons, appears at every precursor of the modulated signal and rapidly attenuates within about 10 ns. In other words, the maximum modulated frequency of this system is about 100 MHz. Such fluctuations can be suppressed by using advanced PIN diodes [40]. Hence, both ASK and PSK can be manipulated by a single digital designer SP system to two independent frequency channels introduced by the discrete dispersion engineering.

## DISCUSSION

Introducing the state-change materials, the digital designer SPs break the barrier between digital science and plasmonic. Dynamic manipulation of the plasmon state can be achieved by tuning the environment of the state-change materials, and thus the resonance of EM fields around the designer SPs can be customized as well. Outstanding discrete dispersion control and digital modulation performances on the proposed designer SP device are demonstrated analytically and experimentally. With adapted manufacturing technology of waveguide and state-change medium, there will be no substantive challenge for the digital designer SPs to be applied in the terahertz regime, implying a promising prospect of both plasmonic and digital system research.

## METHODS

### Substrate material

In numerical simulations and real experiments, the substrate material is selected as Rogers RT5880, which is a kind of high-quality printed circuit board (PCB) commonly used in the microwave and millimeter-wave frequencies. Previous research has demonstrated that Rogers RT5880 has an almost non-dispersive real part of the permittivity (Re *ε_r_* = 2.2) and a very small imaginary part (Im *ε_r_* = 0.002 @10 GHz), which allows us to ignore the substrate loss so as to simplify the analysis. More detailed information on Rogers RT5880 can be found in the relevant datasheet [41].

### The ASK and PSK modulation

The modulated transmission signals in the case of a monochromatic incident wave and square wave signal modulation can be expressed analytically as

(1) \begin{eqnarray*} {T|}_{f_{\rm {inc}}}&=&{{A}_0(t)|}_{f_{\rm {inc}}}\left[\sum \limits_{n=-\infty}^{\infty }R(t- nT)\right]\nonumber\\ &+&{{A}_1(t)|}_{f_{\rm {inc}}}\left[1\!-\!\sum \limits_{n=-\infty}^{\infty }R(t- nT)\right] \end{eqnarray*}

in which *A_i_*(t) ${\vert}$_*f*inc_ is the corresponding function for ‘*i*’ (*i* = 0 or 1) state designer SPs at the incident frequency *f*_inc_. Meanwhile, *T* is the modulation period, and the function *R(t)* is the square wave, which is defined by

(2) \begin{equation*}R(t)=\left\{\begin{array}{c}0\\ {}1\\ {}0\end{array}\begin{array}{c}\\ {}\\ {}\end{array}\begin{array}{c}\\ {}\\ {}\end{array}\begin{array}{c}t < 0\\ {}0 \lt t \lt \alpha T\\ {}t \gt \alpha {T} \end{array}\right.\end{equation*}

where *α* is the duty ratio of the modulation signal.

For the PSK modulation case, the modulated transmitted signals *T*_low_ can be expressed as

(3) \begin{eqnarray*} {T}_{\mathit {low}}&=&\beta {e}^{i\left(2\pi {f}_{\rm {inc}}t+{\varphi}_0\right)}\sum \limits_{n=-\infty}^{\infty }R\left(t- nT\right)\nonumber\\ &+&{e}^{i\left(2\pi {f}_{\rm {inc}}t+{\varphi}_0+\pi \right)}\left[1-\sum \limits_{n=-\infty}^{\infty }R\left(t- nT\right)\right]\nonumber\\ \end{eqnarray*}

and its Fourier transform can also be written as

(4) \begin{eqnarray*} {F}_{\mathit {low}}&=&{e}^{i{\varphi}_0}{e}^{- ik\pi \alpha}\left[\sum \limits_{k=-\infty}^{\infty}\left(\beta \frac{\sin \left( k\pi \alpha \right)}{k\pi}\right.\right.\nonumber\\ &-&\left.\left.\!\!\frac{\sin \left( k\pi \left(1-\alpha \right)\right)}{k\pi}{e}^{- ik\pi}\right){e}^{i2\pi \left[{f}_{\rm {inc}}+k/T\right]t}\right].\nonumber\\ \end{eqnarray*}

Furthermore, its *k*th-order harmonic spectral coefficient *FL_k_* can be expressed as

(5) \begin{equation*} {\mathit {FL}}_k=\beta \frac{\sin \left( k\pi \alpha \right)}{k\pi}-{\left(-1\right)}^{k+1}\frac{\sin \left( k\pi \left(1-\alpha \right)\right)}{k\pi}. \end{equation*}

On the other hands, for the ASK case, the modulated transmitted signals *T*_high_ can be expressed as

(6) \begin{equation*} {T}_{\mathit {high}}={e}^{i\left(2\pi {f}_{\rm {inc}}t+{\varphi}_0\right)}\left[1-\sum \limits_{p=-\infty}^{\infty }R\left(t- pT\right)\right]\!. \end{equation*}

Further, we rewrite it based on the Fourier transform:

(7) \begin{eqnarray*} {F}_{\mathit {high}}&=&\left[{e}^{i{\varphi}_0}\sum \limits_{k=-\infty}^{\infty}\frac{\sin \left( k\pi \left(1-\alpha \right)\right)}{k\pi}\right.\nonumber\\ &\times& \left.\vphantom{\sum \limits_{k=-\infty}^{\infty}}{e}^{- ik\pi \left(1+\alpha \right)}{e}^{i2\pi \left[{f}_{\rm inc}+k/T\right]\mathrm{t}}\right] \end{eqnarray*}

and its *k*th-order harmonic spectral coefficient *FH_k_* can be expressed as

(8) \begin{equation*} {\mathit {FH}}_k=\frac{\sin \left( k\pi \left(1-\alpha \right)\right)}{k\pi}. \end{equation*}

### The spectrum measurement system

From microwave to terahertz frequencies, the vector network analyzer (VNA) is a powerful tool to obtain the spectrum information, including the amplitudes and phases of the transmission and reflection coefficients from two ports. Here, we performed all spectrum measurements using an Agilent N5230C, as described in Fig. S4a in the online supporting information. To be compatible with the spectrum measurement system, we weld two standard sub-miniature-A (SMA) type connectors and added them to the two ports of the digital plasmonic waveguide, leading to about 1 dB insertion loss.

### The non-linear spectrum/time domain measurement system

Because VNA is a piece of linear measurement equipment, a non-linear spectrum measurement cannot be supported by the above spectrum measurement system. Hence, the non-linear spectrum measurement is implemented by a new measurement system, which is composed of a sinusoidal wave generator (Keysight M9393A PXIe) to generate the incident wave, a computer generating the digital code, and a spectrometer applied as a receiver, as shown in Fig. S4b in SI. If we need the time-domain spectrum, we only need to replace the receiver with a high-performance oscilloscope (Keysight DSOZ254A Infiniium), which can cover the main frequency band of the digital SP devices.

### The near-field measurement system

The near-field measurement was implemented by a near-electric-field mapper, which was composed of a VNA (Agilent N5230C), a monopole antenna as the detector, and a planar platform that can move in the *xoy* plane under the control of a stepper motor. Port 1 of the VNA was connected to the input of the digital plasmonic waveguide, while port 2 of the VNA was connected to the monopole antenna, which was fixed 0.5 mm above the digital plasmonic waveguide, to detect the vertical (*z*) components of the electric fields, as shown in Fig. S4c in SI.

## Supplementary Material

nwz148_Supplemental_File
