# Supplementary material for: Active digital spoof plasmonics
Source: Natl Sci Rev. 2019 Oct 4;7(2):261–9. doi: 10.1093/nsr/nwz148 (PMC8288847; doi:10.1093/nsr/nwz148)
Supplement: nwz148_Supplemental_File [file nwz148_supplemental_file.docx]

Supplementary Information for

**Active digital spoof plasmonics**

Hao Chi Zhang^1, 2, †^, Tie Jun Cui^1, †,^ **^*^**, Yu Luo^2, †,^ **^*^**, Jingjing Zhang^1, 2, †^, Jie Xu^1^,

Pei Hang He^1^, Le Peng Zhang^1^

^1^ State Key Laboratory of Millimeter Waves, Southeast University, Nanjing 210096, China

^2^ The Photonics Institute and Centre for OptoElectronics and Biophotonics, School of Electrical and Electronic Engineering, Nanyang Technological University, Nanyang Avenue, 639798, Singapore

† These authors contribute equally to this work.

**^*^** E-mail: tjcui@seu.edu.cn, luoyu@ntu.edu.sg

This PDF file includes:

Materials and Methods

Supplementary Figure 1. The schematic diagram of simulated setting of Eigen-mode solver.

Supplementary Figure 2. The geometrical schematic diagram of digital SPP structure.

Supplementary Figure 3. The zoom-in view of PSK modulation.

Supplementary Figure 4. The schematic diagrams of measurement systems for spectra, nonlinear spectra and near fields.

Supplementary Table 1. The geometrical parameters of the ambient circuits.

1. **Eigen-mode** **simulation of dispersion curves**

The simulation is conducted by the Eigen-mode solver of the commercial software, CST Microwave Studio. Since this solver do not support the open boundary, we use the electric and magnetic boundaries around the designer SPs structure, and the distance between the structure and boundary is set as 100 mm which is about 3 times of the wavelength of the central frequency. In addition, periodic boundary with sweeping phase difference is set on the two sections of designer SPs in the transmission direction, shown in Fig. S1.


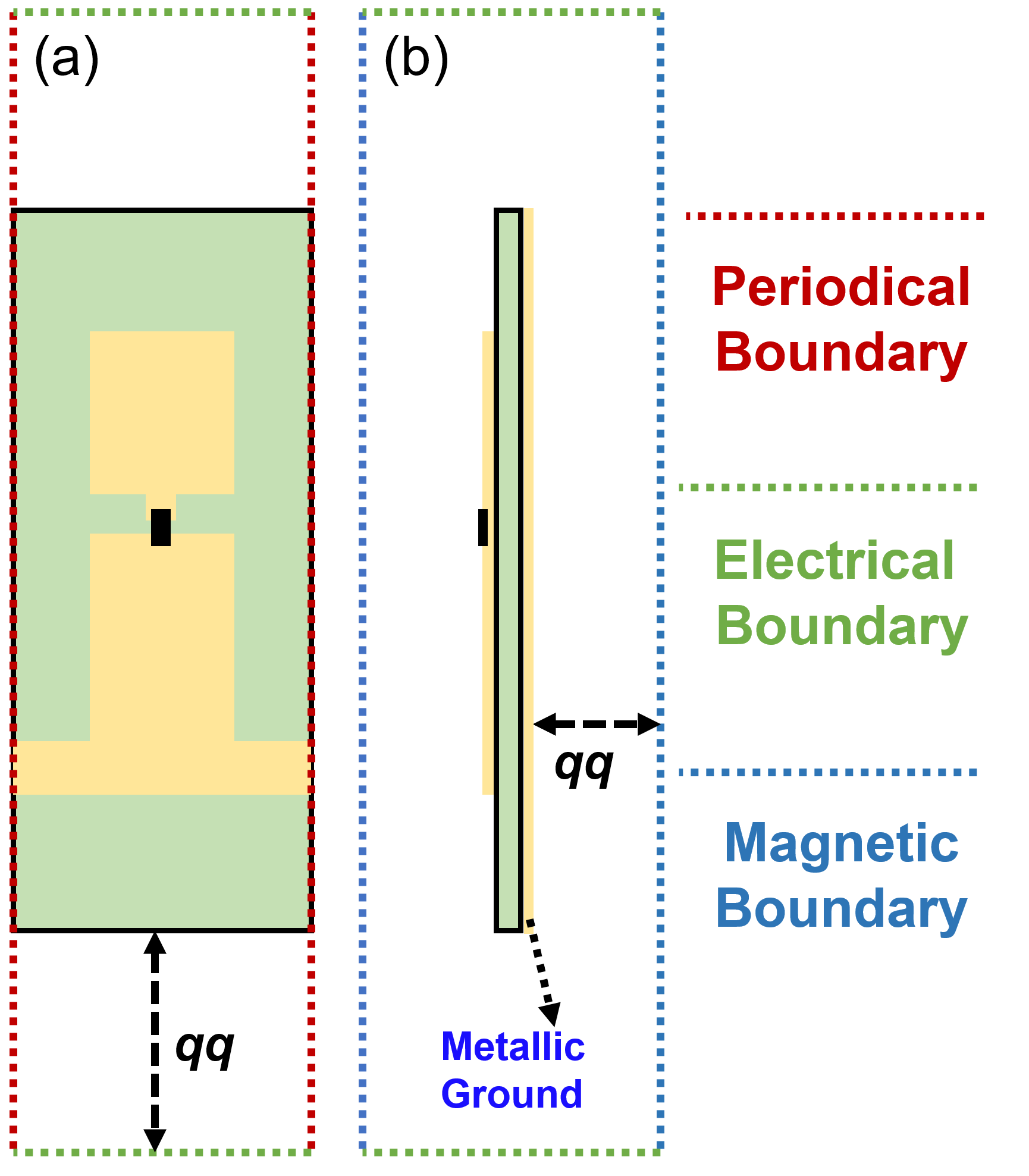


**Fig. S1.** **The schematic diagram of simulated setting of Eigen-mode solver.** (a) the front view of the simulated setting. (b) the lateral view of the simulated setting.

1. **Detailed geometrical parameters of the designer SPP structure**

In this Supplementary Information (SI), we provide the detailed geometrical parameters to show the design of the digital SPP structure, as sketched in Fig. S1. Please note that the P point in Fig. S1b and Q point in Fig. S1c are the mapping of P and Q in Fig. S1a. Fig. S1a displays the design of integrating the state-change medium with the metallic structure, in which the slit *s_2_* and width *w_2_* of the metallic patch are chosen as 0.25 mm and 0.3 mm, respectively, and the length *s_3_*, width *w_3_* and thickness *t_3_* (not shown in Fig. S1a) of the state-change medium are chosen as 0.3 mm, 0.2 mm and 0.2 mm, respectively. In order to introduce the digital code bias signals into system and stop the high-frequency energy leaking to bias signal ports, the designed ambient circuits composed of series microstrip inductors, parallel capacitors and a resistor. The whole circuit behaves like a low-pass filter (LPF) is used. Here, for convenience, the LPF is designed based on the series inductance and parallel grounded capacitance. However, considering the fact that lumped inductance cannot be used at that frequency due to parasitic effect of packaging, we replaced the lumped inductance using the meander line in the design of LPF, as shown in Fig. S1b. And the detailed parameters can be found in Table S1. Finally, the smooth convector based on gradient index structure is shown in Fig. S1c, in which the length *l_5_* and depth *w_5_* are chosen as 12.8 mm and 1 mm, respectively.

**
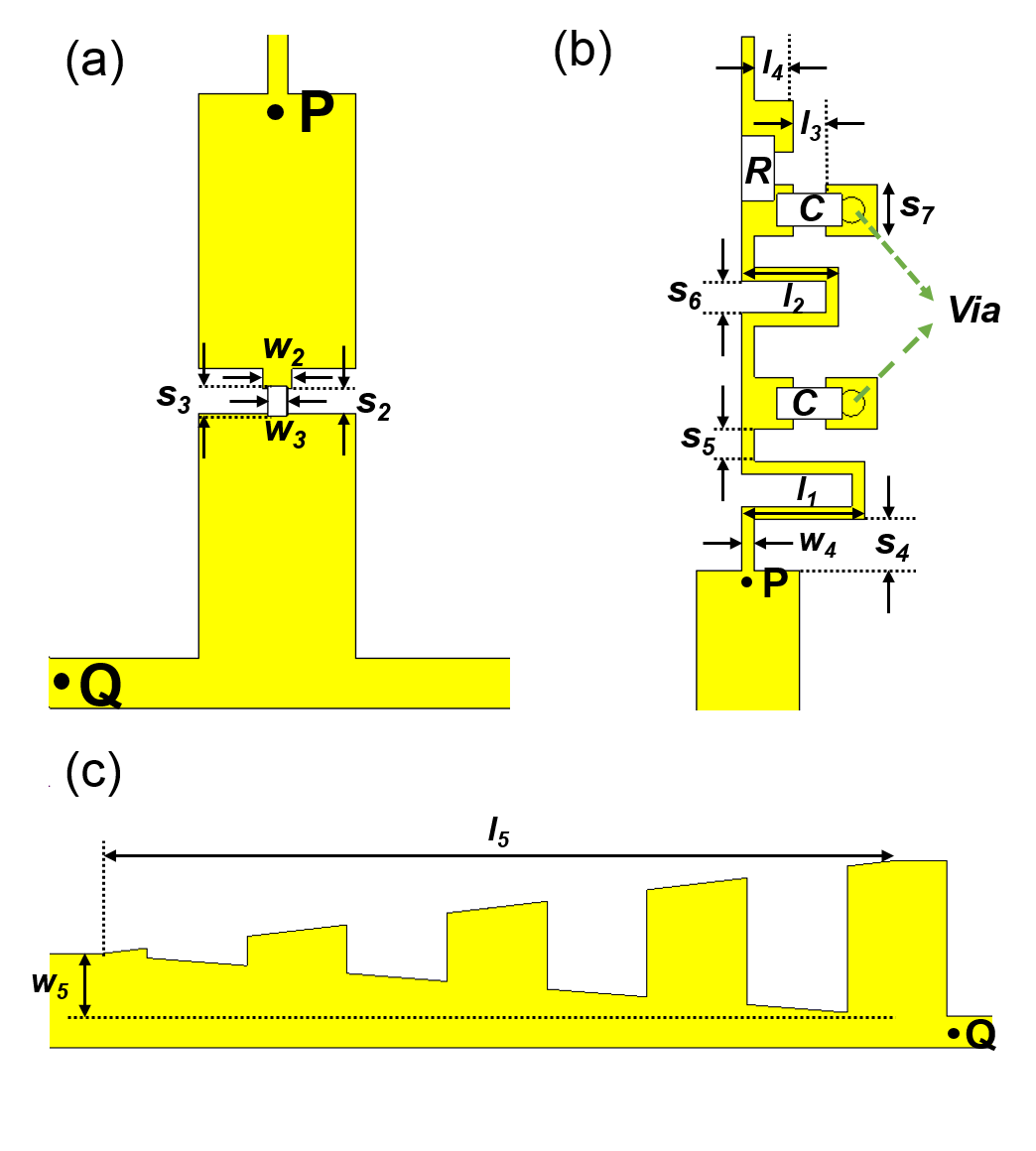
**

**Fig. S2. The geometrical schematic diagram of digital SPP structure.** (a) The geometrical schematic diagram of a single unit, in which the slit *s_2_* and width *w_2_* of the metallic patch are chosen as 0.25 mm and 0.3 mm, respectively, and the length *s_3_*, width *w_3_* and thickness *t_3_* (not shown in Fig. S1a) of the state-change medium are given as 0.3 mm, 0.2 mm and 0.2 mm, respectively. (b) The geometrical schematic diagram of a single ambient circuit and the detailed parameters can be found in Table S1. (c) The geometrical schematic diagram of gradient index structure, in which the length *l_5_* and depth *w_5_* are chosen as 12.8 mm and 1 mm, respectively.

**Table S1. The geometrical parameters of the ambient circuits**

| *R* | C | *s_4_* | *s_5_* |
| --- | --- | --- | --- |
| 600 ohm | 1 pF | 0.8 mm | 0.5 mm |
| *s_6_* | *s_7_* | *l_1_* | *l_2_* |
| 0.5 mm | 0.8 mm | 1.9 mm | 1.5 mm |
| *l_3_* | *l_4_* | *w_4_* | _ |
| 0.5 mm | 0.6 mm | - 1. mm | _ |

1. **Zoom-in view of PSK modulation**

**
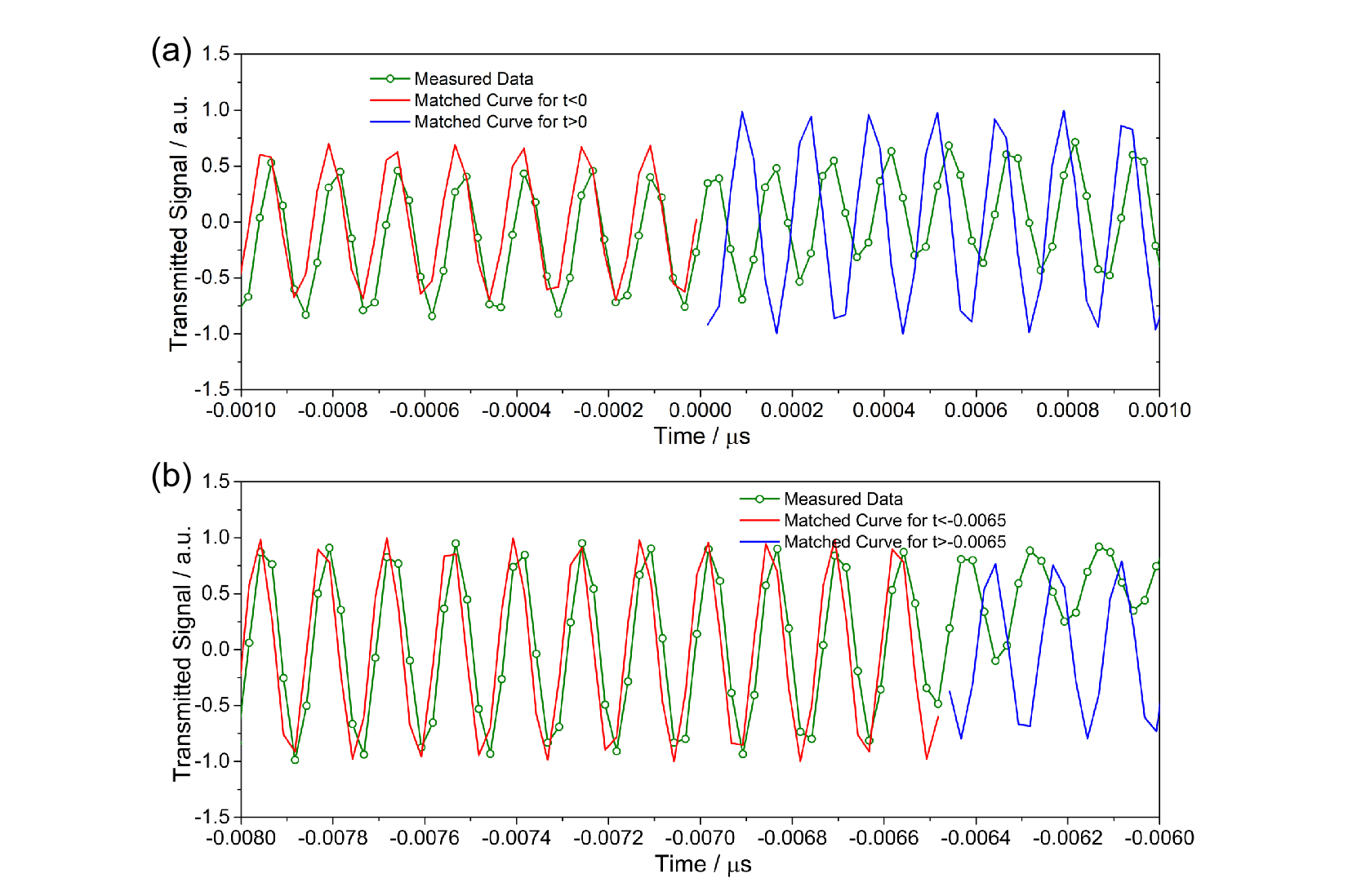
**

**Fig. S3. The zoom-in view of PSK modulation.** (a) Zoom-in view of PSK modulation of Fig. 4(c). (b) Zoom-in view of PSK modulation of Fig. 4(f). The reason why phase step change cannot be observed is that the [charge and discharge](E:/Dict/8.5.3.0/resultui/html/index.html#/javascript:;) of the state-change medium cannot be achieved instantaneously.

1. **The schematic diagrams of measurement systems**

**
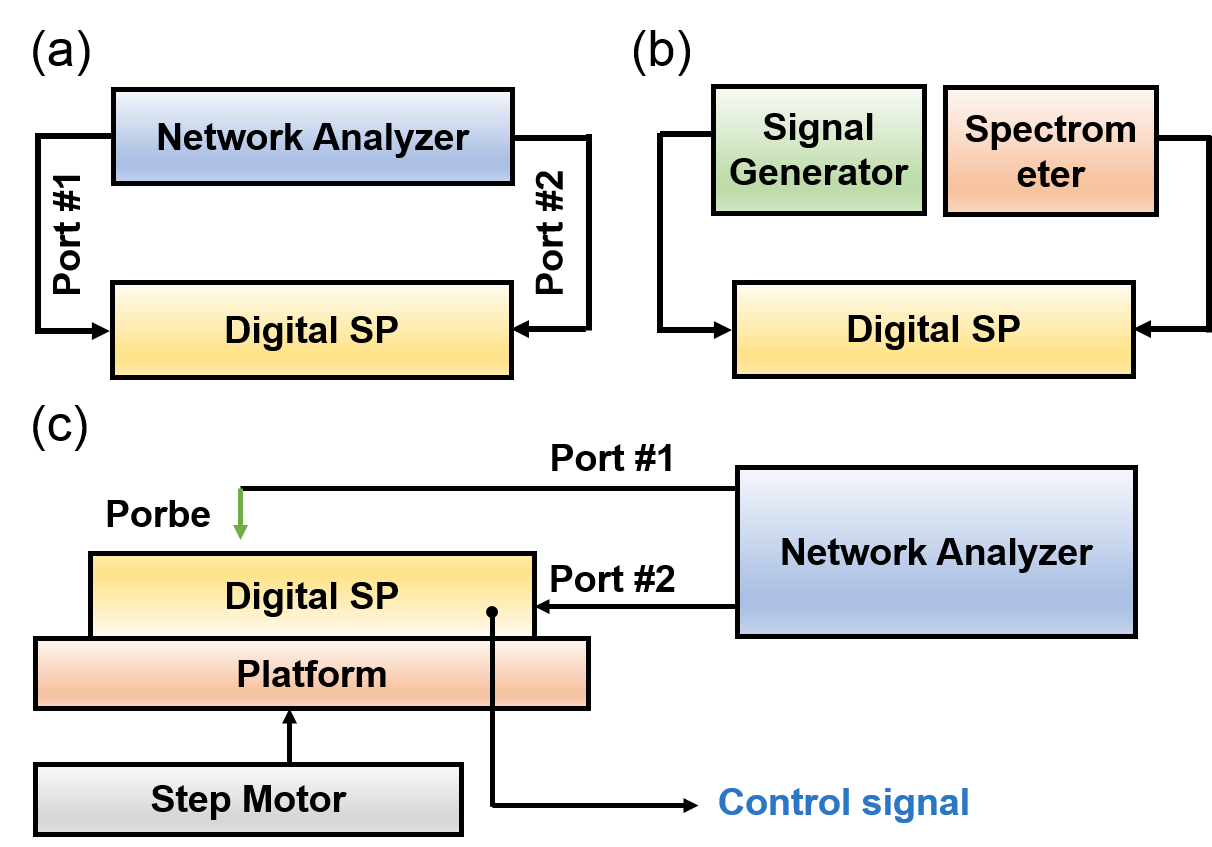
**

**Fig. S4. The schematic diagrams of measurement systems for spectra， nonlinear spectra and near fields.** (a) The schematic diagram for spectrum measurement. (b) The schematic diagram for nonlinear spectrum measurement. (c) The schematic diagram for near-field measurement.
